# Musculoskeletal pain in older adults at the end-of-life: a systematic search and critical review of the literature with priorities for future research

**DOI:** 10.1186/1472-684X-12-27

**Published:** 2013-07-25

**Authors:** Alison Kate Lillie, Sue Read, Christian Mallen, Peter Croft, John McBeth

**Affiliations:** 1 Keele University School of Nursing and Midwifery, Clinical Education Centre, University Hospital of North Staffordshire, Stoke on Trent, ST4 6QG, Staffordshire, UK; 2 Keele University, Research Institute for Primary Care & Health Sciences, Keele University, Keele, ST5 5BG, Staffordshire, UK

**Keywords:** Palliative, Pain, Musculoskeletal, End of life, Systematic review

## Abstract

**Background:**

Pain is an important issue in end of life care. Although musculoskeletal pain is common in older adults, it is rarely associated with the cause of death and may be overlooked as death approaches. Hence a major target for improving quality of life may be being missed.

**Methods:**

The aim of this study was to systematically search and critically review the literature on musculoskeletal pain at the end of life. Amed, Cinahl, Internurse, Medline, Psych Info, Web of Knowledge and Cochrane review databases were searched for relevant research up to September 2012. The search strategy combined key words expanding the terms ‘palliative’ for population, ‘musculoskeletal’ for exposure, and ‘pain’ for outcome. Predefined inclusion and exclusion criteria were applied.

**Results:**

Five relevant papers and one letter to the editor were found, including case studies and epidemiological research. Current evidence suggests musculoskeletal pain is common in older adults at the end of life and that it can have a substantial impact on individual experience. No information about community based treatment of musculoskeletal pain at the end of life was found.

**Conclusion:**

Priorities for future research include high quality epidemiological studies to establish the prevalence, natural history, impact, assessment, patient priorities and outcomes associated with musculoskeletal pain in the end of life period, and intervention research that provides an evidence base for treatment.

## Background

Pain has been described as a more terrible lord of mankind than even death itself [1]; nevertheless it is known that many people die with unnecessary pain [2]. Musculoskeletal pain is a common symptom that is frequently under-reported and inadequately treated in older adults [3], the stage of life when most people die [4]. Musculoskeletal pain has the potential to impact on end of life care, especially as many of the first line strategies promoted, including exercise and self-management [5] may not be applicable or appropriate as death approaches [6]. The rationale driving this paper is that the most common cause of pain in older people [7] may be being overlooked as it is rarely implicated as a cause of death, despite the potential for musculoskeletal disease to be a substantial cause of pain and discomfort in the dying person.

Musculoskeletal pain derives from a pathophysiologically diverse set of musculoskeletal conditions [8] including osteoarthritis, rheumatoid arthritis and spinal trouble. It is commonly classified according to pain location (hip, knee, lower back) although most people with chronic pain have pain at multiple sites [9]. One reason the topic has remained largely unexamined is that most studies of pain prevalence in the elderly are cross sectional and provide no information about the progression of pain with time [7,10]. Most studies of pain and other symptoms at the end of life consider the needs of people with a specific advancing progressive disease [11-13], and do not include symptoms associated with co-morbid diseases like arthritis [12], or other common causes of musculoskeletal pain. This is compounded by the dearth of research to inform the treatment of pain in the elderly [5,14]. A recent review of pain management found no well-designed studies of analgesia that specifically focused on elderly patients requiring palliative care [15].

Another reason for the lack of research in this area may be that musculoskeletal pains are frequently considered to be part of the normal ‘wear and tear’ of aging [5]. For instance, Klinkenberg et al [16] compared the agreement between the reporting of symptoms and disease by elderly patients (n = 270) in research interviews, with proxy reporting in after-death interviews with significant others and after-death questionnaires completed by General Practitioners (GPs). Osteoarthritis (OA) was the chronic disease with the lowest concordance between both patient and proxy report and between patient and GP report, with patients reporting much higher prevalence in both comparisons. Klinkenberg et al [16] suggested that the reason for the poor concordance was that healthcare professionals and significant others were more likely to recall the illness that led to death than a chronic disease that was integrated into a patients daily life.

The prevalence of musculoskeletal pain is known to increase with age until it stabilises around age 65 [17]. However, the prevalence of disabling pain that impacts on life increases notably among older people into the oldest age-groups [18]. The impact on individuals can be significant [3]. A review of chronic pain prevalence in older people found estimates ranging from 18-57% [19]. The wide range was partly explained by the variation in definitions used for chronic pain [19]. There is less precise information about the prevalence, impact or treatment of musculoskeletal pain at the end of life. Consequently it is possible that a major cause of pain is being overlooked and a potential target for improving quality of life is being ignored. The objective of this study was to conduct a systematic search of the literature with the aim of highlighting what is currently known about musculoskeletal pain in older adults at the end of life and the identification of priorities for future research.

## Methods

### Search strategy

A modified PICO search [18] was used to identify information regarding musculoskeletal pain at the end of life. No comparison group was included as a scoping search had shown that there was limited literature available and we therefore planned to keep the search parameters as broad as possible. The key words used to define the population were ‘palliative’, ‘end of life’, ‘death and dying’, ‘terminal care’ or ‘terminally ill’. ‘Musculoskeletal’, ‘arthritis’, ‘osteoarthritis’ or ‘rheumatoid’ were used to define the exposure whilst ‘pain’, ‘arthralgia’ or ‘polyarthralgia’ were used to define outcome. The databases searched were Amed, Cinahl, Internurse, Medline, PsychInfo,and Web of Knowledge (from inception to September 2012). (See Table 1 for further details). As Internurse had a more limited search function it was searched separately. The Cochrane database was searched but no relevant review was found. The grey literature was searched using http://www.opengrey.eu. The reference lists of all relevant research papers found were searched for further citations. Independent advice about the search strategy was obtained from an information specialist.

**Table 1:** Search process

**PICO term**	**Expanded search term**	**Medline**	**Web of knowledge**	**PsychInfo**	**Cinahl**	**Ahmed**
P	‘Palliative’ or ‘end of life’ or ‘terminal’ or ‘death and dying’	813,136	4 443,679	20,030	29,261	478,730
I	‘Arthralgia’ or ‘polyarthralgia’ or ‘pain’	416,896	1,171,198	26,285	133,016	677,575
O	‘Arthritis’ or ‘rheumatoid’ or ‘osteoarthritis’ or ‘musculoskeletal’	123,750	7,413,381	64,124	33,391	199,139
P + I + O	Terms combined with ‘AND’	1383	2192	85	41	236

### Study selection

Inclusion criteria were that papers must be written in English and report original research that considered adults aged 50 or older. There is no general agreement about when old age begins [20]. This search used the broad definition of 50+ as used by the World Health Organisation [20], both to maximise the potential literature found and to acknowledge that socially constructed concepts of age often include biological as well as chronological factors. (However, no papers were found which had to be excluded because they only focused on younger adults). Papers that highlighted pain as a diagnostic feature of disease with the aim of delaying or preventing death were excluded. Similarly studies about the effect of pain on mortality and studies that documented the clinical course of musculoskeletal diseases were excluded. Finally paediatric, microbiological, animal and cadaveric research was also excluded.

**Figure 1:**
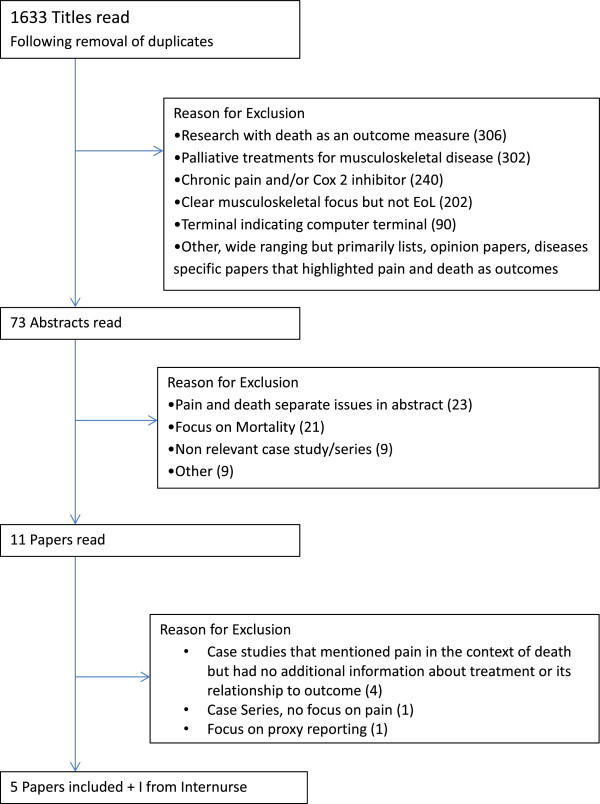
Selection of Included Studies.

Refworks web based bibliographical management software was used to assist study selection. Identified studies from all databases (except Internurse) were combined. Following the removal of duplicate papers 1633 remained. Figure 1 summarizes the selection process. All papers were initially sorted by title. The abstracts of papers were read when the paper appeared relevant from the title or when it was unclear from the title if the paper was relevant (73 abstracts read). If the abstract suggested that there was original research about musculoskeletal pain at end of life, the paper was read (12 papers). These twelve papers were read by a second person to independently validate the inclusion criteria. Four relevant papers and one ‘letter to the editor’ were included in this review. They comprised of three case studies and two epidemiological studies. One paper, a case study [21], located through Internurse was also included making a total of six relevant studies in the review.

### Quality assessment

Quality assessment of research is important to assess trustworthiness [22]. However, eligibility criteria were deliberately kept broad to maximise the information available. Although case studies are generally considered to provide a weak level of evidence they do provide valid and useful information about complex clinical situations [22]. They also alert practitioners to rare side effects and benefits of disease and treatments [23,24]. Hence they were included in this review. Despite the importance of critical appraisal no research was excluded on the basis of quality assessment. Due to the diversity of impetuses behind the papers a standard proforma was not used to extract data, rather relevant facts were extracted though multiple readings of the papers. Case study data is summarised in Table 2 and epidemiological studies in Table 3.

**Table 2:** Study characteristics and key findings: case studies

**Author**	**Study aim**	**Study population**	**Key findings**
Lewin (2012)	To discuss percutaneous cervical cordotomy for non-cancer pain	Gentleman (67) with cancer and RA.	Percutaneous cervical cordotomy gave effective pain relief for last 11 months of life
		Persistent, severe, right hip pain limited quality of life (QoL)	
Katz et al. (2008)	To describe arthroplasty for MSK pain at EoL	Older woman with metastatic lung cancer & lymphoma. QoL severely limited by hip OA	Surgery successful; complete relief of hip pain. Increased mobility enabling independent living for last year of life
Del Fabbro et al. (2007)	To discuss temporary palliative sedation	Woman (60’s) with lung cancer and limited metastatic disease. Chronic osteoporosis, OA and chronic back pain	Uncontrolled severe pain despite opioid rotation (oral morphine equivalent of 1440mgs in 24 hrs). Temporary palliative sedation used with good effect (Patient able to return home). Long term chronic musculoskeletal pain (+ somatisation, depression and terminal illness) contributed to complex symptom control requirements at EoL.
		Intractable lumber pain	
Greenstreet (2001)	To discuss concept of total pain	Woman (early 50s) with colon cancer, metastatic lung disease & PE. History of OA and bilateral knee arthroplasty. Significant pain in left knee from chronic osteomyelitis Pain exacerbated by movement	Treatment complex required. Including, high dose morphine sulphate (460mgs SR 12hrly) + Corticosteroids used as adjuvant to reduce inflammation of the knee. + IV antibiotics to promote comfort
			Non pharmacological measures included a brace to immobilise knee joint, crutches to reduce weight baring and ensuring leg supported and elevated

**Table 3:** Study characteristics and key findings: epidemiological studies

**Paper**	**Smith et al. (2010)**	**Borgsteede et al. (2007)**
Study Aim	To describe the epidemiology of pain at the EoL	To describe the prevalence of symptoms in patients receiving palliative care at home
Study Population	Data obtained from The Health and Retirement Study (USA)	Study nested in the 2nd Dutch National Survey of General Practice (DNSGP-2)
Sampling Frame	National probability sample of US households	A representative sample of 104 Dutch GPs
Sample Population	Community dwelling older adults who died within 24 months of final period of data collection (N = 4703)	Patients who died with an observation period of at least 3 months in the survey year and were labelled as palliative care patients by their GP (N = 429)
Data Collection	Telephone interviews (and some home visits)	From GP records of GP/Patients encounters
Sample Characteristics	Male = 52.3% Female 47.7%	Male 47%, Female 53%
	Mean age, (SD): 75.7, (10.8)	Mean age (SD): 76.8 (13.9)
	Age distribution: 21% < 65; 24% > = 66–75: 36% > = 67–85:19% > 86	Age distribution: 28% < 70; 24% = 70–79: 31% = 80–89: 16% > 90
	Terminal Diagnosis: Cancer 27.6%; Heart Disease 29.7%: Frailty 11.8%; Sudden Death 16.7%; Other 14.2% (62.2% had arthritis)	Terminal Diagnosis: Cancer 56%; Heart failure 11%; COPD 3%; Other disease 25%; Multiple non cancer diseases 5%
	Key measure: Prevalence	Key measure: Prevalence
Key Findings	Arthritis strongly associated with pain at EoL (*P* < 0.001).	The prevalence of musculoskeletal symptoms was 20% in patient physician encounters.
	In final month of life pain prevalence was 60% in people with arthritis versus 26% in people without arthritis.	

## Results

### Case reports

Lewin et al’s [25] letter described the use of cervical cordotomy for musculoskeletal pain in a 67 year old man with metastatic oesophageal cancer and rheumatoid arthritis (RA). Following chemotherapy he had persistent, severe right hip and buttock pain at the site of an earlier total hip replacement, which restricted mobility. As he responded poorly to opioids and had a prognosis of less than a year the cordotomy was performed enabling the patient to mobilise independently till he died eleven months later.

Katz et al [26] discussed the case of an elderly woman with lymphoma and a non-small cell lung cancer. Her main symptom was pain due to advanced left hip OA. This severely restricted her ability to mobilise. Her pain had been treated with physiotherapy and ibuprofen with minimal effect. She had a total hip replacement following a full discussion of the enhanced risks of surgery. Within a week she had complete relief from her left hip pain and regained full mobility following rehabilitation. This enabled her to spend most of her final year of life living independently.

Del Fabbro et al [27] discussed an unusually complex case of a woman in her sixties with lung cancer with limited metastatic disease and a history of osteoporosis, OA, and chronic back pain. She was admitted to the palliative care unit with intractable pain that was poorly controlled using intravenous (IV) opioids (oral morphine equivalence of up to of 1600mgs daily). The main focus of the paper is on the temporary palliative sedation that was used to control delirium and enable assessment of symptom severity whilst rotating opioids to maximise analgesic affect with minimum side effects, enabling discharge home for a period of weeks before death.

This case highlighted how the treatment of long term chronic musculoskeletal pain may have inadvertently and adversely affected the care needs as death approached. The woman had been receiving muscle relaxants and opioid analgesia for chronic back pain since the death of her husband. The possibility that she had somatised her grief and depression during her bereavement is discussed. It is suggested that this maladaptive coping mechanism of requesting opioids for existential distress as well as physical pain, contributed to the rapid escalation of opioids that led to delirium and the necessity of temporary sedation [27].

Greenstreet [21] focused on ‘Hannah’: a woman in her early 50s with colon cancer, metastatic lung disease and a pulmonary embolism (PE). She had a history of OA and bilateral knee arthroplasty. The main physical symptom was pain in the left knee due to osteomyelitis. Hannah was not fit for surgery and non-steroidal anti-inflammatory medication was inappropriate due to the risk of haemorrhage as she was prescribed anticoagulant medication following her PE. Corticosteroids and a course of intravenous antibiotics were prescribed with the aim of reducing the inflammation, and associated pain, caused by the osteomyelitis. Analgesia was given in accordance with the WHO Cancer Pain Ladder [28] and a strong opioid (morphine) was gradually titrated until a good analgesic effect was achieved at rest. This was realised with 460 mg slow release morphine twice daily. Breakthrough pain, commonly provoked through movement remained. Non pharmacological measures to reduce these episodes of breakthrough pain included a brace to immobilise the knee joint, crutches to minimise weight bearing, and ensuring the leg was elevated when Hannah was sitting. Psychological support, massage and aromatherapy were also used to reduce pain perception.

### Epidemiological papers

Smith et al [29] considered the epidemiology of pain during the last two years of life. Information was obtained from 4703 decedents (mean age 76), from The Health and Retirement study, a longitudinal cohort study of community dwelling adults living in the USA. Taking the last interview before death participants were placed into one of twenty four cohorts on the basis of the number of months between interview and death and their responses compared with the background prevalence of pain amongst participants of the same age who did not die.

The authors found that the presence of arthritis was strongly associated with pain at the end of life. The prevalence of pain in the last month of life was 60% of people with arthritis versus 26% among people without arthritis (*P* <0.001). This did not differ by terminal disease category, nor was there any evidence for an interaction between arthritis and any terminal disease category [29]. During the two years before death the prevalence of pain remained stable at approximately 40% for people with arthritis and 14% for people without arthritis, until the last four months of life when it increased steadily to the prevalence figures reported above.

Borgsteede et al [30] reported on the prevalence of symptoms in patients receiving palliative care at home. Their study was completed within the framework of a nationwide cross sectional study of general practice in the Netherlands. A representative sample of participating GPs received a questionnaire regarding patients who had received palliative care and died at home. Information was then retrieved from GP records, using the international classification of primary care (ICPC), regarding the GP-patient encounters in the last three months for 429 patients. Symptoms were classified into categories according to ICPC chapters. Musculoskeletal symptoms had a 20% prevalence in patient-physician encounters.

## Discussion

The findings present a dichotomy of methods and focus with two epidemiological papers that suggest that musculoskeletal symptoms have a substantial impact at the end of life in the general population and four cases studies showing that musculoskeletal pain can be a significant issue for individuals requiring unusually sophisticated pain control measures including temporary sedation, cordotomy, arthroplasty and very high dose opiates. No information was found about the way that musculoskeletal symptoms were assessed and treated in the general population. Despite this, the findings do give some indication of the prevalence, impact and treatment of musculoskeletal pain at the end of life.

### Prevalence

The population based studies indicated that musculoskeletal pain is a common and significant issue at the end of life. Smith et al’s [29] study, the first epidemiological study to look at pain at the end of life, draws attention to the fact that musculoskeletal disease may have as much, if not more, effect on whether a person dies in pain than the condition that is the cause of death. Unfortunately, Smith et al [29] do not define what is mean by the term ‘arthritis’. This is important as prevalence estimates of musculoskeletal disease occurrence can vary considerably depending on the phenotype definition [31]. Also 19% of Smith et al’s [29] data was obtained by proxies who may have underreported musculoskeletal symptoms [16].

Smith et al’s [29] study used the time between interview and death to document a significant increase in pain prevalence in people with arthritis as death approached. The authors highlighted the limitations of using cross sectional data in this fashion. Despite this, the findings emphasise the need to be especially vigilant for pain in people with co-morbid musculoskeletal disease in the final months of life [32]. Borgsteede et al [30] supported this by showing that musculoskeletal symptoms were prevalent in at least 20% of patient-GP encounters during the last three months of life. This is higher than the 14% annual prevalence of GP consultations for musculoskeletal disease in the general population reported by Jordan et al [33]. However, the studies were undertaken in different countries and used different systems for classifying consultation data making direct comparison difficult. Furthermore, Borgsteede et al [30] gave no information about the nature or severity of the symptoms, nor does it discuss how, or whether, they were successfully managed in practice. Borgsteed et al [30] suggested that their study may have underestimated the prevalence of musculoskeletal symptoms as GP’s were unlikely to register all the symptoms affecting patients at the end of life and the records represented the most important symptoms as perceived by the GPs, rather than documenting the patients perspective [30]. Smith et al [29] may also have a systematic bias underestimating the true prevalence of musculoskeletal pain. The health and retirement study excluded people living in institutions, and admission to care homes is commonly prompted by reduced physical functioning [34].

Although both population based studies found musculoskeletal disease had a significant impact at the end of life, the prevalence of symptoms recorded varied significantly: 60% in Smith et al [29] and 20% in Borgsteede et al [30]. As Smith et al [29] does not discuss how ‘arthritis’ was defined and Borgsteede et al [30] do not discuss the nature of the musculoskeletal symptoms, comparison is difficult. The extent of the disparity is similar to that observed when estimates of musculoskeletal pain from population surveys are compared with estimates derived from coded primary care data, with surveys consistently suggesting that only a minority of people raise the issue of even severe musculoskeletal pain with their GP [35]. Nevertheless the fact that these figures do not more closely correspond provides tentative initial support for the idea that musculoskeletal pain is common at the end of life, but underestimated as a cause of pain by healthcare professionals. However, an alternative explanation is that raising an issue in a GP consultation is a proxy for severity causing patients to request treatment. Even the lower figure of 20% prevalence suggests that large numbers of people may be significantly affected by musculoskeletal pain. Neither Borgsteede et al [30] or Smith et al [29] were specifically investigating musculoskeletal pain at the end of life and both papers reported that the levels of musculoskeletal pain were new findings that had not been highlighted in previous end of life care research. This emphasises the need for more population based epidemiological studies which specifically focus on musculoskeletal symptoms. This is discussed further below.

### Impact

The four case studies clearly demonstrated that musculoskeletal pain can significantly impact on individuals in diverse ways emphasizing the needs for individualised assessment and treatment of musculoskeletal pain at the end of life. However, as three of these studies describe particularly complex situations it is not possible to extrapolate any information about the impact of musculoskeletal pain to the general population. However the importance of the case histories as illustration is that they highlight that rational treatment targeted at comorbid musculoskeletal pain is a potentially important component of all patients in pain nearing the end of life: they powerfully challenge the assumption that pain in this period should simply be attributed to the condition causing death without considering other concurrent explanations. Neither of the population based studies discussed the impact or treatment of musculoskeletal pain.

### Treatment

Only one of the case studies, Katz et al [26], argued that the treatment described; (total joint replacement), could offer a potent and systematic treatment strategy in the palliative care of patients with advancing progressive disease and concomitant musculoskeletal pain. There was a dearth of studies about the treatments for musculoskeletal pain at the end of life in a primary care setting. This is an important omission because, although most people die in a hospital setting, the majority of the last year of life is lived in the community, either at home or within a care home [2,36].

A possible reason for the lack of information about treatment is that either the standard tools advocated by palliative care, or the treatments advocated for chronic musculoskeletal pain, are effective. Palliative care promotes the use of the World Health Organisation cancer pain ladder [28] for systematic and effective pain management. Although there have been some studies that consider the effectiveness of this tool for cancer pain [37,38], there appears to be no study that considers whether this is an effective way to manage musculoskeletal pain at the end of life. There are, indeed, significant limitations in the evidence base for the use of opioids in chronic musculoskeletal pain [39-41] and the side effects of opiates meant they were ineffective in two of the case reports [25,27]. Furthermore, there are concerns regarding the use of opiates for short term incident or episodic pains [42], like the breakthrough pain provoked by movement described by Greenstreet [21].

The fact that Smith et al [29] reported that the prevalence of pain was similar across the different categories of terminal illness but substantially greater for people with concomitant arthritis, alongside the substantial body of evidence that many older adults live with chronic pain associated with musculoskeletal disease [3,7] tentatively suggests that musculoskeletal pain is not being effectively treated at the end of life. Rather, it is being overlooked as a potentially common cause of pain whilst attention is focused on supporting symptoms associated with concurrent advancing progressive disease.

## Priorities for future research

The limited literature identified emphasises the need for more research into almost every aspect of this topic. However, it is suggested that the three key priorities for future research are:

1) Research that denotes the prevalence, natural history, causes, outcomes, and other factors associated with musculoskeletal pain at the end of life.

More epidemiological research that is specifically designed to focus on the factors that influence the prevalence of musculoskeletal pain at the end of life is needed. Studies should use core standard definitions of musculoskeletal pain to allow comparisons between different studies and enable meta-analysis of results [43]. In particular a longitudinal cohort study of people with musculoskeletal disease would help identify key factors that influence the prevalence of musculoskeletal pain as death approaches. Epidemiological research would also help differentiate the effect of psychosocial factors and treatment factors that influence the experience of pain at the end of life.

2) Research that describes the impact of musculoskeletal pain on older adults at the end of life.

Qualitative research, with different groups of older adults, including the frail elderly, would help elucidate how musculoskeletal pain affects the options and choices available at the end of life. This is particularly important as many of the symptoms associated with musculoskeletal disease are also commonly associated with other advancing progressive incurable disease [11-13]. As musculoskeletal disease can be overlooked at this time [16] more information would help elucidate whether musculoskeletal pain is a significant factor in the end of life experience of the elderly.

3) Research that provides an evidence base for treatment of musculoskeletal pain at the end of life.

Research is needed to document how musculoskeletal pain is being treated at the end of life and which treatments are most effective. Studies that consider the treatment given in a primary care setting are a particularly priority since much of the last year of life is lived in the community, either at home or within a care home [2,35]. Pharmacological and non-pharmacological treatments that are known to be efficacious in the short term like intra-articular injections and topical NSAIDs [44] may be worth re-examining as short acting analgesic measures are frequently valuable at the end of life. Case series that rigorously and systematically describe the effect of treatment on a consecutive series of patients with musculoskeletal pain at the end of life should be instigated. For instance, a case series looking at arthroscopy, as described by Katz et al [26], would demonstrate whether the procedure was clinically acceptable and feasible for a range of patients. Finally, there is a need for randomised controlled trials to ensure that the treatments are effective and safe within a general population.

## Conclusion

This systematic search of the literature suggests that musculoskeletal disease is an important issue that can significantly impact on pain in the elderly at the end of life. It highlights the high prevalence of musculoskeletal symptoms at the end of life and the need for frequent assessment of musculoskeletal pain as death approaches. However, it also draws attention to the dearth of literature regarding evidence based treatment for people dying with musculoskeletal pain. One reason for the previous oversight of this important topic may be that chronic disease that is assimilated into a patient’s daily life is less likely to be the focus of concern than a concomitant advancing progressive disease [16]. Priorities for research include epidemiology studies of musculoskeletal pain at the end of life and its impact on individuals, together with qualitative research into patient priorities related to this topic and research designed to provide an evidence base for treatment at this time.

## Competing interests

The authors declare that there are no conflicts of interest.

## Authors’ contributions

AKL, SR, CM, PC & JM participated in the planning and design of the project. AKL and JM developed the search strategy. AKL performed the search. PC independently read papers to validate the inclusion criteria. AKL, SR, CM, PC and JM drafted the manuscript. All authors read and approved the final manuscript.

## Pre-publication history

The pre-publication history for this paper can be accessed here:

http://www.biomedcentral.com/1472-684X/12/27/prepub
